# Selfitis among Young Individuals in Kyrgyzstan: A Cross‐Sectional Study of Social Media Use and Selfie‐Taking Behaviour

**DOI:** 10.1002/hsr2.72808

**Published:** 2026-07-14

**Authors:** Niyazi Ayhan

**Affiliations:** ^1^ Faculty of Communication Kyrgyz‐Turkish Manas University Bishkek Kyrgyzstan

## Abstract

**Background and Aims:**

This study examines selfie behaviour scores among young individuals in Kyrgyzstan and analyses their association with social media use duration and selfie‐taking behaviour.

**Methods:**

A cross‐sectional survey was conducted in Bishkek, Kyrgyzstan, between January 3 and March 20, 2026. Data were collected from 300 participants aged 18–25 through face‐to‐face questionnaires. Selfitis behaviour was measured using the Selfitis Behavior Scale. Data were analysed using SPSS with t‐test, ANOVA, Pearson correlation, and multiple linear regression.

**Results:**

Selfitis behaviour scores did not differ significantly by gender, age group, or place of residence. Significant differences were found in daily time spent on social media and selfie‐taking frequency. Selfie‐taking frequency showed a stronger association with selfitis behaviour scores. The regression model was statistically significant but had limited explanatory power, accounting for 8.6% of the variance.

**Conclusion:**

Selfie behaviour scores among young adults in Kyrgyzstan were modestly associated with behavioural indicators, particularly selfie‐taking frequency and, to a lesser extent, daily duration of social media use. Given the cross‐sectional design, convenience sampling, self‐report data, and low explained variance, the findings should be interpreted as preliminary associations rather than causal, clinical, or intervention‐related evidence.

## Summary

**What is known:**

- Selfie behaviour is associated with self‐presentation and social media use among young individuals.

**What is new:**

- Selfie‐taking frequency showed a stronger association with selfitis behaviour scores than daily social media use duration.

**Implications:**

- Future research should examine behavioural indicators alongside usage duration before drawing intervention, education, or policy implications**.**

## 1 Introduction

Among young individuals, visibility on social media is not limited to simply having an online presence; it is also an important means for individuals to be noticed, approved of, and gain social acceptance from their peers [1, 2, 3]. In particular, selfie behaviour has become one of the ways young individuals present themselves in the digital environment. In this context, the act of taking and sharing selfies has been emphasised in many studies not only as an individual preference but also as a behaviour intertwined with peer expectations, social norms, and online feedback processes [1, 4, 5]. The need for social acceptance remains particularly salient during young adulthood, alongside the pursuit of online visibility [6, 7, 8]. Therefore, this need may play a dominant role in the prominence of visual sharing behaviours on social media [9]. Yau and Reich [4], Tian et al. [10], and Ullah et al. [11] state that self‐presentation in social media environments is an important process that shapes young individuals' social positions and peer relationships. According to self‐presentation theory, one of the theories explaining this situation, individuals tend to regulate their behaviour in order to create a certain impression on others in social environments [12]. Social comparison theory, on the other hand, suggests that individuals tend to compare themselves with others to evaluate themselves, and that this process becomes more pronounced, especially in visibility‐based environments [13]. Based on all this, selfie behaviour offers an important research area for understanding young individuals' pursuit of digital visibility and their online interaction patterns. In this context, selfie production can be considered not only an individual media activity but also a part of social interaction processes.

Selfie behaviour is often not simply a form of sharing. The intensity of social media use, visual‐sharing habits, and expectations for online interaction largely shape it. Previous research has shown that increased social media use is associated with more frequent selfie‐taking, editing, and sharing behaviours [14, 15, 16]. However, viewing the selfie‐producing process as a single‐step action is often inaccurate. The selfie production process consists of successive stages, including selecting the photo, retaking it, editing, and sharing [16, 17]. The rise in visually based social media use, especially among young individuals, makes photo production and sharing a significant part of social interaction [18]. Therefore, selfie behaviour needs to be considered alongside broader social media usage patterns. Although selfie‐taking is a common digital practice among young individuals, repetitive and excessive selfie‐related behaviours may develop into what the literature describes as selfitis.

While taking and sharing selfies is a common practice of digital visibility among young individuals, in some individuals, this behaviour becomes more frequent, repetitive, and sometimes problematic [19, 20, 21]. At this point, one concept that has gained increasing attention in the literature is selfitis behaviour. Selfitis behaviour is defined not merely as casual sharing, but as a behavioural pattern characterised by increasing frequency, regular repetition, and the expectation of social feedback [22]. In the present study, selfitis behaviour is not treated as a clinical diagnosis or a pathological condition. Instead, it is operationally defined as a self‐reported, continuous research variable measured through the Selfitis Behavior Scale. Accordingly, the selfitis behaviour score refers to participants' scale‐based responses reflecting repetitive selfie‐related behavioural tendencies, rather than to a diagnostic category. This distinction is important because ordinary selfie‐taking, frequent selfie‐taking, self‐presentation, social approval seeking, narcissistic tendencies, and problematic social media use may overlap conceptually. However, they should not be treated as identical constructs. Research shows that the tendency towards selfitis behaviour is closely related to the intensity of social media use, the desire for online visibility, and feedback mechanisms such as likes or comments [16, 23]. This behaviour often develops within a process consisting of stages such as repeatedly taking photos, editing the image, and monitoring feedback after sharing [17]. Some researchers also emphasise that repetitive selfie behaviours are closely related to the need for social validation and online self‐presentation [24]. In this respect, selfitis behaviour differs from casual selfie use and points to a more persistent behavioural pattern. In addition, selfitis behaviour should not be considered equivalent to constructs such as narcissism, self‐esteem, or problematic social media use. Although these constructs may be theoretically related, selfie behaviour specifically refers to repetitive selfie‐related behavioural tendencies associated with digital self‐presentation and online feedback processes [22].

Several studies have indicated that selfie behaviour is not merely a technical media activity, but also constitutes a psychosocial space related to an individual's self‐perception, social sensitivity, and well‐being [25, 26]. Studies with similar samples have also highlighted that self‐presentation through social media during young adulthood can influence an individual's self‐evaluation [27]. Therefore, it would be more accurate to consider selfitis behaviour not simply as the habit of taking frequent selfies, but as a more complex behavioural pattern arising at the intersection of digital self‐presentation, social feedback, and social comparison processes. The current literature has mostly examined selfie‐related behaviours in the context of body image, online self‐presentation, and problematic social media use [25, 28, 29]. However, studies examining selfitis behaviour alongside everyday digital practices, such as time spent on social media, selfie‐taking frequency, and sharing habits, are limited [23, 30]. Furthermore, most research has been conducted in Western societies, and findings on how young adults' social media usage patterns are shaped in different cultural contexts are limited [31]. This suggests that selfie behaviour may not manifest in the same way across different socio‐cultural contexts.

Empirical studies focusing on young individuals' social media use patterns in Central Asian countries, particularly their digital self‐presentation practices such as selfies, appear to be limited. However, it is noted that in societies undergoing rapid digitalisation, such as Kyrgyzstan, the relationship that young people establish with social media may exhibit dynamics different from those in Western contexts due to the influence of the socio‐cultural context [32, 33]. Indeed, studies conducted in Kyrgyzstan reveal that young people's experiences in digital environments are shaped by their level of digital literacy, parental supervision, and socio‐cultural factors [34, 35, 36]. However, the fact that a significant portion of existing studies focus on university students [37, 38] increases the need for research examining how selfie behaviour emerges in young adults. Therefore, considering the relationship between selfie behaviour among young individuals and social media use duration, selfie frequency, and demographic characteristics has the potential to fill a significant gap in the literature. In this context, the present study aimed to examine selfie behaviour among young individuals in Kyrgyzstan within the framework of social media use patterns and specific demographic variables.

The conceptual framework of this study links social media use duration and selfie‐taking frequency to selfitis behaviour through visibility‐oriented self‐presentation and social comparison processes. Drawing on self‐presentation theory and social comparison theory, this study assumes that these behavioural variables may be associated with selfitis behaviour because they reflect young individuals' digital visibility practices and feedback‐seeking behaviours [12, 13, 23]. Accordingly, this study investigated how young adults' scores on selfitis behaviour varied across time spent on social media, selfie‐taking frequency, selfie‐sharing habits, and gender. The following research questions were addressed in this study.

What are the self‐itis behaviour scores among young individuals in Kyrgyzstan?

Do selfie behaviour scores differ across variables such as gender, social media use duration, selfie‐taking frequency, selfie‐sharing frequency, and place of residence (rural/urban)?

To what extent do the duration of social media use and selfie‐taking frequency predict selfitis behaviour scores among young individuals?

## 2 Methods

### 2.1 Research Design

This study was designed using a quantitative, cross‐sectional, correlational survey model to examine the relationships among selfitis behaviour scores, duration of social media use, and selfie‐taking behaviour among young individuals. The quantitative approach was preferred because it allows for the statistical testing of relationships between variables [39, 40].

The research has a cross‐sectional design, collecting data at a single point in time. This design allows for the description of existing behavioural patterns but limits the ability to make causal inferences [41].

The study was also structured within a correlational model framework. In this context, the selfitis behaviour score was the dependent variable, while social media use duration and selfie‐taking frequency were independent variables. The relationships between the variables were tested using correlation and multiple linear regression analyses [42].

The study has a relational and predictive analytical focus. However, because of its cross‐sectional design, the analyses were used to examine statistical associations and predictive patterns rather than causal explanations. This framework is theoretically informed by Goffman's [12] approach to self‐presentation, but the study does not directly test identity construction or impression management mechanisms.

### 2.2 Participants

The participants in this study were young adults living in Bishkek, aged 18 to 25. Data were collected through face‐to‐face surveys conducted on university campuses, in parks, and in other public spaces in Bishkek, using convenience sampling to ensure practical accessibility and direct reach to the target audience. The study sample consisted of 300 participants (*N* = 300).

Participation in the study was entirely voluntary. Inclusion criteria were being between 18 and 25 years of age and actively using social media. Participants who completed the survey form incompletely or incorrectly were excluded from the analysis.

A post‐hoc power analysis was conducted using G*Power 3.1. Based on the observed effect size (f^2^ = 0.094), an alpha level of 0.05, two predictors, and a total sample size of 300, the achieved statistical power was 0.99. This result indicates that the sample size was sufficient to detect the observed effects in the regression model.

### 2.3 Data Collection Tools

#### 2.3.1 Selfitis Behavior Scale

To determine participants' selfitis behaviour scores the Selfitis Behavior Scale developed by Balakrishnan and Griffiths [22] was used. This scale evaluates the behaviours of individuals who take, edit, and share selfies as a repetitive behavioural pattern associated with social feedback processes [22]. The scale consists of 20 items and is answered with a 5‐point Likert scale (1 = strongly disagree, 5 = strongly agree). Higher scores on the scale indicate a higher tendency towards selfitis behaviour.

The scale was adapted into Russian for the research. Forward and back translation methods were used in the translation process; it was carried out by two language experts competent in translation, and the resulting versions were compared to maintain the semantic integrity of the scale. Accordingly, the final form of the scale was created. The necessary permission was obtained from the developers to use the scale in the research.

The total selfitis behaviour score was calculated using the mean score of all scale items. Higher mean scores indicate stronger tendencies towards self‐reported selfitis behaviour. No reverse‐coded items were included in the final scoring process.

### 2.4 Demographic Information Form

A demographic information form developed by the researcher was used. This form includes basic sociodemographic characteristics of participants, such as age, gender, and people they live with, as well as behavioural variables related to the frequency of social media use, taking selfies, and sharing selfies.

Variables related to social media use and selfie behaviour were measured with ordinal categorical response options to reflect usage intensity. These variables were appropriately coded and included in the statistical models during the analysis.

Ordinal variables related to social media use and selfie‐taking frequency were numerically coded prior to analysis. For the selfie‐taking frequency variable, lower numerical values represented higher selfie frequency (1 = daily, 5 = never). Therefore, negative correlation and regression coefficients involving this variable were interpreted in the reverse direction.

### 2.5 Data Collection Process

Data were collected through face‐to‐face questionnaires administered using convenience sampling in Bishkek, Kyrgyzstan, between January 3, 2026, and March 20, 2026. Participants were informed about the purpose of the study, and participation was entirely voluntary. Participants were explicitly informed that they could withdraw from the study at any time without any negative consequence. Informed consent was obtained from all participants before data collection. No identifying personal information was requested, and the data were collected and analysed anonymously. The study did not involve any clinical intervention, treatment, biological sample collection, or collection of sensitive identifying information. The research was conducted in accordance with the ethical principles stated in the Declaration of Helsinki. A total of 320 questionnaires were administered as part of the research. Before the analysis, all questionnaires were screened for missing responses, incomplete completion, and inconsistent response patterns. Questionnaires with missing responses on the main study variables or clearly incomplete forms were excluded from the dataset. After this screening process, 300 valid questionnaires were retained for analysis.

### 2.6 Data Analysis

Research data were analysed using the SPSS 23 statistical package program. Before starting the analyses, the suitability of the data set for parametric tests was evaluated. In this context, the skewness (skewness = 0.495) and kurtosis (kurtosis = −0.547) values related to the total selfitis behaviour score were examined, and it was determined that these values were within the acceptable range. These findings show that the data distribution is normal and that parametric tests are applicable [40, 42].

To determine the reliability of the measurement instrument, Cronbach's alpha was calculated, yielding α = 0.927, which indicated high internal consistency. A principal components analysis was conducted using principal components extraction with Varimax rotation to examine the structure of the Selfitis Behavior Scale. Components with eigenvalues greater than 1 were retained. Item loadings of 0.40 and above were considered meaningful, and cross‐loadings were evaluated when an item loaded at 0.40 or above on more than one component. The Kaiser–Meyer–Olkin value indicated that the sample was suitable for component analysis (KMO = 0.935), and Bartlett's test of sphericity was significant (χ^2^ = 3214.300, df = 190, *p* < 0.001).

The principal components analysis yielded a six‐component structure explaining 72.021% of the total variance. However, several items showed cross‐loadings, which complicated the substantive interpretation of the individual components. Therefore, the components were not interpreted as distinct subscales. Importantly, the presence of cross‐loadings was not treated as evidence of unidimensionality; rather, it indicated that the extracted components were not sufficiently interpretable as separate subscales in the present sample. Given the high internal consistency of the scale and the original use of the Selfitis Behavior Scale as an overall measure of selfitis behaviour, the total mean score was retained for subsequent analyses [22]. This decision is also consistent with previous empirical studies that have treated the Selfitis Behavior Scale as a global measure of selfie‐related behavioural tendencies or have used the overall selfitis score in relation to other psychological and social media‐related variables [43, 44].

Within the scope of descriptive statistics, mean, standard deviation, minimum and maximum values were calculated for selfitis behaviour scores. An independent‐samples t‐test was used to examine whether selfitis behaviour scores differed by gender. One‐way analysis of variance (ANOVA) was used to examine whether selfitis behaviour scores differed across categorical variables, including living partner, childhood caregiver, frequency of social media use, frequency of taking selfies, and frequency of sharing selfies. Post hoc analyses were performed to detect group differences when necessary. Pearson correlation analysis was conducted to examine the relationships between the selfitis behaviour score and the frequency of social media use and of taking selfies. This analysis evaluated the direction and strength of linear relationships between the variables. Multiple linear regression analysis was applied to determine whether social media use duration and selfie‐taking frequency predicted selfitis behaviour scores. In the regression model, selfitis behaviour score was the dependent variable, and frequency of social media use and frequency of taking selfies were the independent variables. The statistical significance level was accepted as *p* < 0.05.

All statistical tests were two‐sided, and the statistical significance level was set at *p* < 0.05. Analyses were conducted using IBM SPSS Statistics version 23.0.

### 2.7 Findings

Table 1 presents the demographic characteristics of the participants (*N* = 300). The gender distribution is balanced: 50.7% of participants are female and 49.3% are male. This indicates that there is no gender imbalance in the sample.

**Table 1:** Demographic characteristics of participants (*N* = 300).

Variable	Category	*n*	%
Gender	Female	152	50.7
	Male	148	49.3
Age group	18–19	73	24.3
	20–21	78	26.0
	22–23	82	27.3
	24–25	67	22.3
Place of longest residence	Rural	132	44.0
	Urban	168	56.0
Daily social media usage time	Less than 1 h	12	4.0
	1–3 h	86	28.7
	4–6 h	155	51.7
	7 h or more	47	15.7

When age groups are examined, participants exhibit a relatively balanced distribution, with the highest rate in the 22–23 age group at 27.3%. This finding indicates that the sample primarily consists of individuals in early adulthood and is consistent with the study's target audience.

When the distribution of participants by the place where they have lived the longest is examined, 56.0% live in the city and 44.0% live in the rural area. This shows that the sample has a heterogeneous structure, predominantly urban but also including rural experiences.

When daily social media usage time is examined, it is noteworthy that a significant proportion of participants are heavy social media users. 51.7% of participants reported using social media for 4–6 h a day, while 28.7% reported using it for 1–3 h. In contrast, the percentage of those who use social media to a limited extent is quite low (4.0%). These findings indicate that social media plays a central role in participants' daily lives and provide a suitable context for examining selfie behaviour.

### 2.8 Selfie Behavior Patterns

Table 2 reveals the patterns of participants' selfie behaviour. It is observed that a significant portion of the participants engage in selfie‐taking behaviour infrequently. The prominence of the “rarely” (44.0%) and “never” (11.7%) options indicates that while selfie behaviour is common in the sample, it is not a widespread practice.

**Table 2:** Selfie behavioural patterns of participants (*N* = 300).

Variable	Category	*n*	%
Selfie taking frequency	Daily	28	9.3
	Several times a week	62	20.7
	Several times a month	43	14.3
	Rarely	132	44.0
	Never	35	11.7
Importance of receiving approval	Not important	159	53.0
	Slightly important	95	31.7
	Important	33	11.0
	Very important	13	4.3
Most frequently shared content	Selfie	65	21.7
	Group photos	57	19.0
	Daily life (e.g., food, places)	103	34.3
	Hobbies/activities	59	19.7
	Other	14	4.7
	None	2	0.7
Sharing motivation	Communication with friends/family	116	38.7
	Receiving likes/comments	27	9.0
	Work/professional purposes	41	13.7
	Self‐expression	107	35.7
	Other	9	3.0
Photo editing before sharing	Never	76	25.3
	Rarely	97	32.3
	Sometimes	75	25.0
	Often	36	12.0
	Always	16	5.3

When the perception of social approval in selfie sharing is examined, it is noteworthy that more than half of the participants (53.0%) consider approval unimportant. This suggests that the search for external approval cannot always explain selfie behaviour and that individual motivations may also be important.

Regarding shared content types, participants most frequently share daily life content (34.3%), while selfie content has a lower share (21.7%). This finding shows that social media use is not only about self‐presentation but also about sharing daily experiences. When sharing motivations were examined, “communication with friends and family” (38.7%) and “self‐expression” (35.7%) stood out. In contrast, the motivation to receive likes and comments (9.0%) remained low, supporting the idea that social media use is not limited to seeking social approval.

Finally, when photo editing behaviour was evaluated, it was observed that a large portion of the participants performed editing operations at a limited level. The high percentages of “rarely” (32.3%) and “never” (25.3%) options indicate that a tendency towards intense visual manipulation is not dominant.

When these findings are considered together, it becomes clear that participants' selfie behaviours are shaped by distinct usage patterns and motivations rather than a homogeneous structure. This situation shows that selfie behaviour is not only a sharing practice but also part of a broader behavioural pattern related to individuals' psychological tendencies. In this context, examining these behavioural patterns in relation to selfitis behaviour scores provides a clearer basis for interpreting the subsequent analyses.

### 2.9 Selfitis Behaviour Scores Across Demographic and Behavioural Variables

An independent‐samples t‐test was conducted to examine whether selfitis behaviour scores differed by gender. The analysis results showed that there was no statistically significant difference in selfitis behaviour scores between female (X̄ = 2.33, SS = 0.75) and male participants (X̄ = 2.25, SS = 0.93), t(298) = 0.891, *p* = 0.374.

Similarly, there was no significant difference in selfitis behaviour scores between participants based on where they lived the longest (rural vs urban), t(298) = −0.350, *p* = 0.727. The average selfitis behaviour scores of participants living in rural areas (X̄ = 2.27, SS = 0.84) and urban areas (X̄ = 2.31, SS = 0.85) were quite similar.

When selfitis behaviour scores were examined across age groups, no significant difference was found, F(3, 296) = 0.368, *p* = 0.776. This finding shows that selfitis behaviour scores do not change significantly according to age.

In contrast, a significant difference in selfitis behaviour scores was found according to daily social media use time. F(3, 296) = 3.431, *p* = 0.017. When group means were examined, it was seen that the highest mean selfitis behaviour score was observed among participants who used social media for 4–6 h a day (X̄ = 2.40, SS = 0.83), and the lowest mean selfitis behaviour score was observed among those who used social media for less than 1 h (X̄ = 1.69, SS = 0.69). Tukey post‐hoc analysis shows a significant difference between participants who use social media for 4–6 h and those who use it for less than 1 h (*p* = 0.025). No significant difference was found in other group comparisons.

When selfitis behaviour scores were examined by selfie frequency, significant differences were found between groups, F(4, 295) = 7.234, *p* < 0.001. Group means show that selfitis behaviour increases with selfie frequency. The highest mean was observed in participants who took selfies “every day” (X̄ = 2.61, SS = 0.77), and the lowest mean was observed in those who “never” took selfies (X̄ = 1.76, SS = 0.89). Tukey post hoc analysis revealed significant differences, especially between participants who “never” took selfies and all other groups (*p* < 0.05 for all comparisons). In addition, there were significant differences between participants who “rarely” took selfies and groups who took selfies more frequently. These findings show that selfie‐taking frequency is significantly associated with selfitis behaviour scores (Table 3).

**Table 3:** Differences in selfitis behaviour scores across demographic and behavioural variables (*N* = 300).

Independent samples test							
Variable	Group	*N*	Mean	Std. deviation	Std. error	t	Sig. (2‐tailed)
Gender	Female	152	2.3349	.75100	.06091	.891	.374
	Male	148	2.2480	.93095	.07652		
Place of longest residence	Rural	132	2.2727	.83594	.07276	‐.350	.727
	Urban	168	2.3071	.85297	.06581		
ANOVA							
Variable	Group	N	Mean	Std. Deviation	df	F	Sig.
Age	18–19	73	2.3342	.79459	3 296	.368	.776
	20–21	78	2.2064	.84163			
	22–23	82	2.3201	.89294			
	24–25	67	2.3112	.85062			
Daily social media usage time	Less than 1 h	12	1.6917	.68816	3 296	3.431	.017
	1–3 h	86	2.1860	.85032			
	4–6 h	155	2.4016	.82720			
	7 h or more	47	2.2777	.85877			
Selfie taking frequency	Daily	28	2.6089	.77065	4 295	7.234	.000
	Several times a week	62	2.5113	.79869			
	Several times a month	43	2.5105	.77686			
	Rarely	132	2.1913	.81305			
	Never	35	1.7614	.89443			

### 2.10 Relationship Between Selfitis Behaviour Scores and Behavioural Variables

The correlation analysis results show significant relationships between selfitis behaviour scores and behavioural variables. Accordingly, a low‐level positive correlation was found between selfitis behaviour scores and daily social media use time (r = 0.121, *p* < 0.05). This finding indicates that selfitis behaviour scores tend to increase as social media use time increases. A moderate negative correlation was found between selfitis behaviour scores and selfie‐taking frequency (r = −0.273, *p* < 0.01). However, considering the coding method of the selfie frequency variable (higher values represent lower frequency), the direction of this relationship should be interpreted in reverse. Accordingly, selfitis behaviour scores increase as selfie‐taking frequency increases.

In contrast, no significant relationship was found between daily social media use time and the frequency of taking selfies (r = −0.039, *p* > 0.05), (Table 4).

**Table 4:** Correlations between selfitis behaviour scores and behavioural variables (*N* = 300).

Variable	1	2	3
1. Selfitis behaviour score	—		
2. Daily social media usage	.121*	—	
3. Selfie taking frequency	−0.273**	−0.039	—

*Note: p* < 0.05, *p* < 0.01. Pearson correlation coefficients are reported.

Multiple linear regression was used to determine which variables predicted selfitis behaviour scores. The model is generally significant, F(2, 297) = 14.050, *p* < 0.001. The model explained 8.6% of the variance in selfitis behaviour scores (R^2^ = 0.086). When the independent variables are examined, selfie‐taking frequency was a statistically significant predictor of selfitis behaviour scores (β = −0.268, *p* < 0.001). However, this result should be interpreted in light of the model's limited explanatory power (R^2^ = .086).

Daily social media use time was also a significant predictor of selfitis behaviour scores (β = .110, *p* < 0.05). This finding suggests that selfitis behaviour tends to increase as social media use time increases. However, the effect of time spent on social media is weaker than that of selfie frequency (Table 5).

**Table 5:** Multiple regression analysis predicting selfitis behaviour scores (*N* = 300).

Predictor	B	SE B	β	t	*p*	Tolerance	VIF
(Constant)	2.570	0.226	—	11.365	.000	—	—
Daily social media usage	0.124	0.063	0.110	1.989	.048	0.998	1.002
Selfie taking frequency	−0.191	0.039	−0.268	−4.833	.000	0.998	1.002

*Note:* Dependent variable: selfitis behaviour score. All predictors entered simultaneously (Enter method). Model statistics: *R* = 0.294, *R^2^* = 0.086, Adjusted *R^2^* = 0.080, *F*(2, 297) = 14.050, *p* < 0.001.

Furthermore, when the multicollinearity values for the multiple linear regression analysis were examined, it was determined that the tolerance values for all variables were above 0.10 and the VIF values were well below 10. This indicates that there is no multicollinearity problem in the model. Regression diagnostics were also examined. The normality of residuals was assessed using the histogram and normal P–P plot of standardised residuals. At the same time, linearity was evaluated through the scatterplot of standardised residuals against standardised predicted values. These diagnostics did not indicate substantial violations of residual normality or linearity assumptions.

## 3 Discussion

The findings indicate that selfitis behaviour scores did not differ significantly by gender, age group, or place of longest residence. This suggests that, within the present sample of young adults, these demographic variables were not strongly associated with variation in selfitis behaviour scores. This result may be understood in relation to the increasingly widespread and routine nature of digital media use among young people, which can reduce some visible differences in everyday platform engagement across basic demographic groups (Pew Research Centre, 2021 [45]). However, the finding should be interpreted cautiously. The absence of significant demographic differences in this study does not mean that demographic or socio‐cultural factors are irrelevant to selfitis‐related behaviour more broadly. Rather, it indicates that the demographic variables examined here did not show statistically significant differences in this particular sample.

Our study also showed that selfitis behaviour scores differed according to daily time spent on social media. Participants who reported using social media for 4–6 h per day had higher selfitis behaviour scores than those who used it for less than 1 h. In addition, the correlation and regression analyses indicated a small but significant positive association between the duration of social media use and selfitis behaviour scores. However, the limited size of this association suggests that time spent on social media alone is not sufficient to explain selfitis‐related tendencies. This interpretation is consistent with recent studies arguing that the psychological meaning of social media use depends not only on duration, but also on how individuals engage with platforms, what types of content they produce, and what forms of social feedback they encounter [46, 47, 48]. Therefore, our study suggests a modest association between social media use duration and selfitis behaviour scores, rather than providing evidence of a broader behavioural mechanism.

One of the clearest findings of our study concerns the association between selfie‐taking frequency and selfitis behaviour scores. Participants who reported taking selfies more frequently had higher selfie‐taking behaviour scores, and selfie‐taking frequency remained the strongest predictor in the regression model. This result is broadly consistent with previous studies linking selfie‐related behaviours to self‐presentation, social approval seeking, attention‐related motives, and narcissistic tendencies [22, 49, 50]. However, our study does not provide evidence that frequent selfie‐taking causes selfitis behaviour or that it reflects a specific psychological mechanism. Goffman's [12] self‐presentation perspective may offer a useful interpretive background for understanding why selfie practices are socially meaningful. However, the present data do not directly test identity construction or impression‐management processes. Therefore, this finding should be interpreted more modestly as an observed association between selfie‐taking frequency and selfitis behaviour scores among young adults.

When the findings are considered together with the regression analysis, our study shows a statistically significant but limited model. The model explained 8.6% of the variance in selfitis behaviour scores, indicating that selfie‐taking frequency and daily social media use duration account for only a modest part of the outcome. This should be treated as an important limitation in interpreting the findings. Although selfie‐taking frequency showed a stronger association with selfitis behaviour scores than social media use duration, the model's overall explanatory power remains low. Therefore, our findings should not be presented as a comprehensive explanation of selfitis behaviour. Rather, they provide preliminary evidence that certain behavioural indicators, particularly selfie‐taking frequency, are modestly associated with selfitis behaviour scores. This interpretation is consistent with broader discussions in digital media research suggesting that online behaviours are shaped by multiple psychological, relational, and platform‐specific factors rather than by usage duration alone [46, 47, 51]. Future studies should therefore include additional variables such as self‐esteem, narcissistic tendencies, social comparison, need for approval, peer feedback, and platform‐specific engagement patterns.

Taken together, our findings suggest that selfitis behaviour scores among young adults in this sample were more consistently associated with behavioural indicators, particularly selfie‐taking frequency, than with the demographic variables examined. However, these conclusions should remain modest. The findings do not establish causal pathways, clinical significance, or broader identity‐related mechanisms. Instead, they indicate that selfie‐taking frequency and, to a lesser extent, social media use duration are associated with selfitis behaviour scores and may serve as useful variables for future research on selfie‐related digital behaviours.

### 3.1 Limitations and Future Research

Our study should be interpreted in light of several limitations. First, the cross‐sectional design prevents any causal interpretation of the associations between social media use duration, selfie‐taking frequency, and selfitis behaviour scores. Although our findings showed statistically significant relationships, they do not indicate whether frequent selfie‐taking contributes to higher selfitis behaviour scores, whether individuals with higher selfitis‐related tendencies take selfies more frequently, or whether other psychological and contextual factors shape both. Future longitudinal, experimental, or mixed‐method studies are needed to clarify the direction and meaning of these associations.

Second, our study relied on self‐report data, which may be affected by common‐method bias, recall bias, and social desirability bias [52]. Although anonymity was ensured, participants' responses may reflect their perceptions of their own social media use and selfie‐taking practices rather than objectively observed behaviours. This is particularly relevant because both daily social media use duration and selfie‐taking frequency were measured through participant reports. Future studies could strengthen measurement validity by combining self‐report scales with digital trace data, platform‐based analytics, diary methods, or observational approaches.

Another limitation concerns the construct validity of selfitis behaviour. Although the concept of selfitis has been used in previous studies to examine excessive or problematic selfie‐related tendencies, its conceptual boundaries remain debated. In particular, ordinary selfie‐taking, frequent selfie‐taking, self‐presentation, social approval seeking, narcissistic tendencies, and problematic social media use may overlap. However, they should not be treated as identical constructs. For this reason, our study refers to selfitis behaviour scores rather than making clinical, diagnostic, or causal claims about selfitis behaviour. Future research should further test the measurement validity and conceptual distinctiveness of selfitis in different cultural contexts.

Third, our study relied on a convenience sample of young adults in Kyrgyzstan. Although the sample provides useful preliminary evidence from an under‐researched Central Asian context, it cannot be considered representative of all young adults in Kyrgyzstan or other cultural settings. Therefore, the findings should not be generalised beyond the characteristics of the present sample. Future studies should use larger, more diverse, and probability‐based samples to examine whether similar associations are observed across different regions, educational groups, and socio‐cultural contexts.

Finally, the regression model's relatively low explanatory power should be considered a central limitation of our study. The model explained only 8.6% of the variance in selfitis behaviour scores, indicating that selfie‐taking frequency and social media use duration accounted for only a limited portion of the outcome. Therefore, the significant predictors identified in our study should not be interpreted as strong determinants of selfitis behaviour. Future research should include additional psychological, relational, and platform‐related variables, such as narcissistic tendencies, self‐esteem, social comparison processes, need for social approval, peer feedback, and platform‐specific engagement patterns, to develop a more comprehensive understanding of selfitis‐related behaviour among young adults.

## 4 Conclusion

Our study examined the associations between social media use duration, selfie‐taking frequency, and selfitis behaviour scores among young adults in Kyrgyzstan. The findings showed that selfitis behaviour scores did not differ significantly by gender, age group, or place of longest residence. However, daily social media use duration and selfie‐taking frequency were significantly associated with selfitis behaviour scores. Among these variables, selfie‐taking frequency showed the strongest association.

These findings should be interpreted cautiously. The cross‐sectional design, convenience sampling, self‐report data, low explained variance, and unresolved construct validity issues limit the strength of the conclusions that can be drawn. Therefore, our study does not provide evidence of causality, clinical diagnosis, behavioural mechanisms, identity construction, or intervention effects. Rather, it offers preliminary evidence of modest associations between social media use duration, selfie‐taking frequency, and selfitis behaviour scores in a young adult sample.

Future research should examine these relationships using longitudinal, mixed‐method, and culturally comparative designs. Further studies should also clarify the conceptual boundaries between ordinary selfie‐taking, frequent selfie‐taking, self‐presentation, social approval seeking, narcissistic tendencies, problematic social media use, and selfitis‐related behaviour.

## Author Contributions

**Niyazi Ayhan:** conceptualization, methodology, visualization, formal analysis, data curation; resources, writing – review and editing.

## Funding

This research received no specific grant from any funding agency in the public, commercial, or not‐for‐profit sectors. No funding source or financial relationship had any role in the study design, data collection, analysis, interpretation of data, writing of the manuscript, or decision to submit the article for publication.

## Ethics Statement

This study fits with Health Science Reports because it investigates a contemporary behavioral health issue related to social media use and psychological well‐being among young adults. It provides original public health evidence from an underrepresented population using validated measures and appropriate statistical methods. This study was reviewed and approved by the Scientific Research and Publication Ethics Committee of Kyrgyz‐Turkish Manas University, Bishkek, Kyrgyzstan, under decision number 2026.09.002. All participants were adults aged 18–25 and participated voluntarily. Participants were informed about the purpose of the study, the confidentiality of their responses, and their right to withdraw at any time without negative consequences. Informed consent was obtained from all participants before completing the questionnaire. No personally identifiable information was collected, and all responses were analysed anonymously. The study did not involve any clinical intervention, treatment, biological sample collection, or collection of sensitive identifying information.

## Conflicts of Interest

The author declares no conflicts of interest.

## Author Declaration

The author has read and approved the final version of the manuscript. Niyazi Ayhan had full access to all of the data in this study and takes complete responsibility for the integrity of the data and the accuracy of the data analysis.

## Transparency Statement

Niyazi Ayhan affirms that this manuscript is an honest, accurate, and transparent account of the study being reported; that no important aspects of the study have been omitted; and that any discrepancies from the study as planned have been explained.

## Data Availability

The data that support the findings of this study are available on request from the corresponding author. The data are not publicly available due to privacy or ethical restrictions. The data that support the findings of this study are available from the corresponding author upon reasonable request. Due to confidentiality and participant anonymity considerations, the dataset is not publicly available.
